# Beneficial Effects of Spices in Food Preservation and Safety

**DOI:** 10.3389/fmicb.2016.01394

**Published:** 2016-09-21

**Authors:** Davide Gottardi, Danka Bukvicki, Sahdeo Prasad, Amit K. Tyagi

**Affiliations:** ^1^Department of Agricultural and Food Sciences, Alma Mater Studiorum, University of BolognaCesena, Italy; ^2^Faculty of Biology, Institute of Botany and Botanical Garden “Jevremovac”, University of BelgradeBelgrade, Serbia; ^3^Division of Cancer Medicine, Department of Experimental Therapeutics, The University of Texas MD Anderson Cancer CenterHouston, TX, USA

**Keywords:** inflammatory diseases, spices, food preservation, disease prevention, antimicrobial

## Abstract

Spices have been used since ancient times. Although they have been employed mainly as flavoring and coloring agents, their role in food safety and preservation have also been studied *in vitro* and *in vivo*. Spices have exhibited numerous health benefits in preventing and treating a wide variety of diseases such as cancer, aging, metabolic, neurological, cardiovascular, and inflammatory diseases. The present review aims to provide a comprehensive summary of the most relevant and recent findings on spices and their active compounds in terms of targets and mode of action; in particular, their potential use in food preservation and enhancement of shelf life as a natural bioingredient.

## Introduction

Plant, animal, and microbes represent an unlimited source of compounds with medicinal properties (Tajkarimi et al., 2010). Since ancient time, humans are using spices as nutritional agents (Kaefer and Milner, 2008). According to the U.S. Food and Drug Administration (FDA), spice is an “aromatic vegetable substance in the whole, broken, or ground form, the significant function of which in food is seasoning rather than nutrition” and from which “no portion of any volatile oil or other flavoring principle has been removed” (Sung et al., 2012).

More than 100 varieties of spices are produced throughout the world. Asia is the main leader for the production of spices, particularly of cinnamon, pepper, nutmeg, cloves, and ginger, while Europe grows mainly basil, bay leaves, celery leaves, chives, coriander, dill tips, thyme, and watercress. In America, instead, pepper, nutmeg, ginger, allspice, and sesame seed are mainly produced (Prasad et al., 2011).

Although spices have been used (mostly dried seed, fruit, root, bark, or vegetative material) for rituals, cosmetics and perfumery, their flavoring, coloring and, especially, preservative properties have founded wide applications both in the traditional food preparations and in the food industry. In fact, many compounds isolated from spices (Table 1) have shown antimicrobial activity against some of the most common microorganisms that affect the food quality and shelf life (Tajkarimi et al., 2010). The introduction of spices through the meals has various beneficial effects as well. For instance, they can stimulate the secretion of saliva, promote the digestion, prevent from cold and influenza, and reduce nausea and vomiting (Ravindran, 2002; Sultana et al., 2010). In this manuscript we provide an overview on spices and their constituent as a natural food preservatives *in vitro* and *in vivo*.

**Table 1:** **Antimicrobial potential of phytochemicals (spices) for food preservation; *In vitro* study**.

**Scientific/Common name**	**Major compounds**	**Microorganisms/Model**	**References**
1. *Acacia victoriae* (Wattleseed)	Avicin, Saponins	*S. cerevisiae*	Simons et al., 2006
2. *Aframomum melegueta*	Gingerol	*A. niger, Salmonella* spp.,	Nneka and Jude, 2012
(Grains of paradise)		*E. coli*	Juliani et al., 2008
3. *Aframomum corrorima* (Korarima)	1,8-Cineole, Sabinene,	*A. flavus, Penicillium expansum*	Hymete et al., 2006
	Nerolidol	*E. coli, Salmonella* spp.	Eyob et al., 2008
		*Klebsiella* spp.	Doherty et al., 2010
4. *Allium sativum* (Garlic)	Diallyl sulfide, Allicin	*St. aureus*, S. Typhi,	Yadav and Singh, 2004
		*B. cereus, B subtilis*	
		*E. coli, Ls. monocytogenes*,	
5. *Allium schoenoprasum* (Chives)	Allicin, Diallyl sulfides	*E. coli*	Rattanachaikunsopon and Phumkhachorn, 2008
			Shirshova et al., 2013
6. *Alkanna tinctoria* (Alkanet)	Pulegone, 1,8-Cineole,	—–	Ozer et al., 2010
	α-Terpinyl acetate, Isophytol,	—–	Prasad et al., 2011
	Alkannin, Shikonin		
7. *Alpinia galanga* (Greater galanga)	Galango-isoflavonoid,	*S.* Typhimurium*, St. aureus*	Kaushik et al., 2011
	β-Sitosterol, Galangin,	*B. subtilis, A. niger*	
	β-Caryophyllene, β-Selinene	*Ls. monocytogenes*	
8. *Amomum subulatum* (Black cardamom)	——-	*E. coli, P. aeruginosa*	Bhatt et al., 2014
9. *Angelica archangelica* (Angelica)	α-Pinene, δ-3-Carene,	*E. coli, St. aureus*	Fraternale et al., 2014
	Limonene, Phellandrene		Rather et al., 2013
10. *Anethum graveolens* (Dill)	Carvone, Limonene,	*Clostridium botulinum*,	Peerakam et al., 2014
	Myristicin, Anethole, Eugenol	*P. aeruginosa*,	Ceylan and Fung, 2004
		*St. aureus, Y. Enterocolitica*	
11. *Apium graveolens* (Celery seed)	β-Pinene, Camphene	*St. aureus, E. coli*	Baananou et al., 2013
	Cumene, Limonene	*P. aeruginosa*	
12. *Armoracia rusticana* (Scherb)	Isothiocyanate, Catechin	*B. subtilis, St. aureus*	Mucete et al., 2006
	Kaempferol, Quercetin,		Prasad et al., 2011
13. *Artemisia dracunculus* (Tarragon)	Artemisinin Phenolic acids	*St. aureus*	Obolskiy et al., 2011
	Coumarins, Flavonoids,	*Ls. monocytogenes*	
		*P. aeruginosa*	
14. *Boesenbergia rotunda* (Fingerroot)	Pinostrobin, Pinocembrin,	*Ls. monocytogenes*	Eng-Chong et al., 2012
	Cardamonin, Boesenbergin A	*B. cereus, St. aureus*	
	Boesenbergin B	*Lactobacillus plantarum*	
	Camphor, Linalool, Camphene	*L. cellobiosus, C. albicans*	
15. *Brassica juncea* (Brown mustard)	Isothiocyanate, Diallyl trisulfide,	*Ls. monocytogenes, St. aureus*	
	Allyl- isothiocyanate	*S. enteritidis, S. veneziana*,	Miceli et al., 2014
		*En. hormaechei, En. cloacae*,	Anuradha et al., 2012
		*Citrobacter freundii, K. pneumoniae*	Sethi et al., 2013
		*En. sakazakii, En. amnigenus*	
16. *Brassica nigra* (Black mustard)	Gallic acid, Rutin, Caffeic acid	*E. coli, St. aureus*	Bhatia and Sharma, 2012
	Quercetin, Ferulic acid		Rajamurugan et al., 2012
17. *Bunium persicum* (Black cumin)	γ-Terpinene, Cuminaldehyde	*B. subtilis, St. aureus*	Mazidi et al., 2012
	ρ-Cymene, Limonene		Ghderi et al., 2014
18. *Capsicum annuum* (Chilli pepper)	Capsaicin	*St. aureus, S.* Typhimurium	Koffi-Nevry et al., 2012
19. *Carum carvi* (Caraway)	Carvone, Limonene,	*E. coli, P. aeruginosa*	Agrahari and Singh, 2014
	Carvacrol, Anethole		
20. *Cinnamomum aromaticum* (Cassia)	Cinnamaldehyde, Eugenol	*E. coli, S.* Typhimurium	Bansode, 2012
		*Ls. monocytogenes*	
		*P. aeruginosa, S. enteritidis*	Frankova et al., 2014
21. *Cinnamomum burmannii*	Galacturonic acid	*St. aureus, E. coli*	Al-Dhubiab, 2012
(Indonesian cinnamon)	Cinnamyl alcohol, Coumarin	*B. cereus, S. anatum*	
	Cinnamaldehyde	*Ls. monocytogenes*	
22. *Cinnamomum verum* (Cinnamon)	Cinnamic aldehyde, Eugenol		Yadav and Singh, 2004
		*E. coli, Ps. fluorescens*	Unlu et al., 2010
			Naveed et al., 2013
23. *Citrus hystrix* (Kaffir lime)	Limonene, Citronellal,	*E. coli, B. cereus*	Tabassum and Vidzasagar, 2013
	β-Pinene	*St. aureus*	
			Ng et al., 2011
24. *Ceratonia siliqua* (Carob tree)	Nonadecane, Heneicosane	*Ls. monocytogenes*	Hsouna et al., 2011
	Farnesol, Camphor	*B. cereus, St. aureus*	
		*E. coli, P. aeruginosa*	
25. *Citrus aurantifolia* (Lime)	Limonene, β-Pinene	*St. aureus, A. niger*	Pathan et al., 2012
	γ-Terpinene, Citral		Spadaro et al., 2012
26. *Coriandrum sativum* (Coriander)	Dodecenal, 1-Decanol	*S. epidermidis, St. aureus*	Bharti et al., 2012
	Ergosterol	*P. aeruginosa*,	Zhu et al., 2011
27. *Crocus sativus* (Saffron)	Lauric acid, Hexadecanoic acid,	*E. coli, B. subtilis*	Sethi et al., 2013
	4-Hydroxy dihydro-	*Ps. fluorescens, St. aureus*	Zheng et al., 2011
	-2(3H)-furanone,	*C. freundii*	Bhargava, 2011
	Stigmasterol, Crocetin, Crocin		
28. *Curcuma longa* (Turmeric)	Curcumin	S. Typhi*, Ls. monocytogenes*	Moghadamtousi et al., 2014
		*Clostridium* spp.	Radwan et al., 2014
		*St. aureus, E. coli, B. cereus*,	
		*B. subtilis, C. albicans*,	
		*Y. enterocolitica, P. notatum*,	
		*S. cerevisiae*	
29. *Cuminum cyminum* (Cumin)	Cuminal	*B. cereus, B. subtilis*,	Ceylan and Fung, 2004
		*Ls. monocytogenes*,	Jirovetz et al., 2005
		*C. freundii, K. pneumoniae*	Sethi et al., 2013
		*Ps. fluorescens*,	
		*S. enteritidis, St. aureus*	
		*A. niger, S. cerevisiae*	
		*C. albicans*	
30. *Cymbopogon citrates* (Lemon grass)	Citral, Myrcene, Linalool,	*E. coli, C. albicans*,	Prasad et al., 2011
	Farnesol		Tyagi and Malik, 2010b
			Vazirian et al., 2012
31. *Elettaria cardamomum*	1,8-Cineole, Linalool	*B. cereus, Ls. monocytogenes*	Savan and Kucukbay, 2013
(Green cardamom)	α-Terpinyl acetate	*St. aureus, S. enteritidis*	Malti et al., 2007
		*P. aeruginosa*	
32. *Eruca sativa* (Rocket)	Erucic acid, Oleic acid	*S. aureus, S. epidermidis*	Gulfraz et al., 2011
		*P. aeruginosa*	
33. *Eryngium foetidum*	E-2-Dodecenal (“eryngial”)	*St. aureus, B. subtilis*	Shavandi et al., 2012
(Long coriander)	Dodecanoic acid	*Ls. monocytogenes*	Ngang et al., 2014
			Sharon et al., 2007
34. *Ferula asafetida*	α-Pinene, α-Terpineol, Azulene	*E. coli, B. subtilis*	Mahendra and Bisht, 2012
(Asafoetida)		*P. chrysogenum, A. ochraceus*	Divya et al., 2014
35. *Foeniculum vulgare* (Fennel)	Anethole	*B. cereus, S. enteritidis*,	Ceylan and Fung, 2004
		*Y. enterocolitica*	Shahat et al., 2011
		*St. aureus, B. subtilis*	
		*E. coli, P. aeruginosa*	
		*A. niger, C. vulgaris*	
		*Shigella dysenteriae, E. coli*	
36. *Garcinia indica* (Kokum)	Garcinol	*E. coli, B. cereus*	Elumalai and Eswaraiah, 2011
		*St. aureus, C. albicans*	
37. *Heracleum persicum* (Golpar)	Pimpinellin, Isopimpinellin	*C. albicans*	Hemati et al., 2010
	Bergapten, Isobergapten	*St. aureus*	
38. *Hyssopus officinalis* (Hyssop)	Isopinocamphone, Terpinen-4-ol	*E. coli, S.* Typhimurium,	Di Pasqua et al., 2005
	Pinocarvone, Carvacrol	*C. albicans, S. aureus*	Süleyman et al., 2010
39. *Houttuynia cordata*	Aristolactams, Houttuynoside A	S. Typhimurium	Kumar et al., 2014
(Chameleon plant)	Quercitrin, Quercetin-3-O-β-D-		
	-galactopyranoside		
40. *Illicium verum* (Star anise)	Shikimic acid, Anethole	*B. cereus*	Shan et al., 2007
41. *Kaempferia galanga* (Kencur)	Ethyl-cinnamate, 1,8-cineole	*St. aureus, E. coli*	Umar et al., 2011
	Camphene, Borneol, Kaempferol	*C. albicans*	
	Kaempferide		
42. *Laurus nobilis* (Bay)	1,8-Cineole, α-Pinene, Limonene	*Alternaria alternata, E. coli*	Xu et al., 2014
	2-Carene		Cherrat et al., 2014
43. *Lavandula angustifolia*	1,8-Cineole, Camphor, Borneole	*St. aureus*	Cavanagh and Wilkinson, 2005
	(Lavender)	*P. aeruginosa, E. coli*	Torabbeigi and Azar, 2013
44. *Limnophila aromatic*	Ocimene, Terpinolene, Camphor	*St. aureus, B. cereus*	Gorai et al., 2014
(Finger grass)		*S. epidermidis*	
45. *Lippia adoensis* (Koseret)	Linalool, Germacrene D	*St. aureus, C. albicans*	Folashade and Egharevba, 2012
		*S. cerevisiae*	
46. *Lippia graveolens*	Thymol, Carvacrol, flavonoids	*M. luteus, Salmonella* spp.	Hernández-Hernández et al., 2014
(Mexican oregano)		*Aspergillus niger*	
		Herpes simplex virus	
		human respiratory syncytial virus	
		and human rotavirus	Pilau et al., 2011
47. *Maranta arundinacea* (Arrowroot)	Flavonoids, terpenoids	*E. coli, Ls. monocytogenes*,	Kim and Fung, 2003
		*S. enteritidis, St. aureus*	Rajashekhara et al., 2013
48. *Melissa officinalis* (Balm)	Neral, Citronellal, Isomenthone,		
	Menthone, β-Caryophyllene,	*Shigella sonnei*	Moradkhani et al., 2010
	Carvacrol		
49. *Mentha piperita* (Mint)	Menthol; 1,8-cineole	*E. coli, P. aeruginosa, St. aureus*,	Sharafi et al., 2010
		*Streptococcus faecalis, C. albicans*	Saharkhiz et al., 2012
			McKay and Blumberg, 2006
			Tyagi et al., 2013
50. *Monodora myristica*	Cymene, α-Phellandrene	*St. aureus, B. cereus*	Owokotomo and Ekundayo, 2012
(Calabash nutmeg)	Germacrene D-4-ol	*C. albicans*	
			Odoh et al., 2004
51. *Murraya koenigii*	Murrayanol		
(Curry leaf)	Murrayacine, Mahanine	*Staphylococus* sp.	Handral et al., 2012
52. *Myrica gale* (Gale)	Cymene, β-Elemene,	*St. aureus, B. subtilis*	Nakata et al., 2013
	Myrcene, Limonene	*S. cerevisiae, C. albicans*	
53. *Myristica fragrans*	Myristicin, Sabinene	*St. aureus, B. subtilis*	Gupta et al., 2013b
(Nutmeg)	β-Pinene	*P. aeruginosa, A. niger*	Radwan et al., 2014
		*Clostridium* spp.	
54. *Myrrhis odorata* (Cicely)	p-Cymene, α-Terpinene,	*E. coli, St. aureus*,	Rancic et al., 2005
	δ-Cadinene	*C. albicans, A. niger*	
55. *Myrtus communis* (Myrtle)	Myrtenyl acetate, 1,8-Cineole,	*Ls. monocytogenes*	Amensour et al., 2010
	α-Pinene	*P. aeruginosa*	Cherrat et al., 2014
56*. Nigella sativa* (Black caraway)	Thymoquinone, Nigellone	*St. aureus*	Islam et al., 2012
		*E. coli, P. aeruginosa*	
57. *Ocimum canum*	α-Terpineol, Chavicol,	Food spoiling bacteria	Vyry Wouatsa et al., 2014
	Chavibetol		
58. *Ocimum basilicum* (Basil)	1,8-Cineole	*B. subtilis, E. coli*,	Moghaddam et al., 2011
	Linalool, Methyl chavicol	*S.* Typhimurium*, S. aureus*	Shirazi et al., 2014
		*Ls. monocytogenes*,	Burt, 2004;
		*Cl. botulinum*	Shirazi et al., 2014
		*Ls. innocua, Ps. fragi*,	Alves-Silva et al., 2013
		*Ps. fluorescens, Yarrowia lipolytica*	
		*C. albicans*	
59. *Olea europaea* (Olive)	Oleuropein	*B. cereus, E. coli*	Faiza et al., 2011
			El and Karakaya, 2009
60. *Olax subscorpioidea*	———–	*C. albicans, C. tropicalis*	Dzoyem et al., 2014
61. *Origanum vulgare* (Oregano)	Carvacrol	*E. coli*,	
		*Ls. monocytogenes*	Siroli et al., 2014b
		*S. cerevisiae*	Lv et al., 2011
		*Ls. monocytogenes*	
62*. Origanum majorana*	———–	*B. subtilis, E. coli*	Leeja and Thopil, 2007
(Marjoram)		*P. aeruginosa, St. aureus*	
		*A. niger*	
63. *Pandanus amaryllifolius*	2-Acetyl-1-pyrroline	*E. coli*	Routray and Rayaguru, 2010
(Pandan leaves)			Faras et al., 2014
64. *Petroselinum crispum*	Kaempferol, Quercetin	*B. cereus, St. aureus*,	Haidaria et al., 2011
(Parsley)		*Ls. monocytogenes*	Shan et al., 2007
65. *Persicaria odorata*	β-Caryophyllene,	*St. aureus, E. coli*	Shavandi et al., 2012
(Vietnamese coriander)	β-Caryophyllene,		Sasongko et al., 2011
	Caryophyllene oxide		
66. *Pimpinella anisum* (Anise)	Anethole	*A. ochraceus*	Krisch et al., 2011
		*Fusarium moniliforme*	
67. *Piper betle* (Betel)	Eugenol, Acetyleugenol	*St. aureus, E. coli*	Prakash et al., 2010
		*Vibrio cholerae*	Hoque et al., 2011
68. *Piper capense* (Timiz)	β-Pinene, Sabinene	*St. aureus*	Woguem et al., 2013
69. *Piper guineense*	Lignans, Amides, Alkaloids,	*St. aureus, E. coli*	Nwinyi et al., 2009
(Ashanti pepper)		Flavonoids, Polyphenols	Juliani et al., 2013
70. *Piper nigrum* (Black peper)	Piperine	*St. aureus, E. coli*	Shiva Rani et al., 2013
		*B. cereus, P. aeruginosa*	
71. *Piper retrofractum*	Piperine	*E. coli, P. aeruginosa*	Khan and Siddiqui, 2007
(Long pepper)		*A. niger*	
72. *Polygonum hydropiper*	Catechin, Polygodial,	*E. coli, B. subtilis*	Moyeenul Huq et al., 2014
(Water-pepper)	Quercetin, Hyperin	*St. aureus*	
		*S. cerevisiae, C. albicans*	
73. *Quassia amara* (Amargo)	Quassin	*E. coli, St. aureus*	Ajaiyeoba and Krebs, 2003
			Cachet et al., 2009
74. *Rhus coriaria* (Sumac)	Quercetin, Myricetin, Kaempferol	*E. coli, St. aureus*	Shabir, 2012
	Gallic acid, Methyl gallate	*Ls. monocytogenes*	
	m-Digallic acid, Ellagic acid		
75. *Rosmarinus officinalis*	*p*-Cymene, Linalool,		Jayasena and Jo, 2013
(Rosemary)	Thymol, γ-Terpinene,	*Brochothrix thermosphacta*	Özcan and Chalchat, 2008
	Carnosic acid, Carnosol	*Pseudomonas* spp.	De La Torre Torres et al., 2015
76. *Ruta graveolens* (Rue)	Rutin	*St. aureus, E. coli*	Hamad, 2012
			Kumar et al., 2014
77. *Salvia officinalis* (Sage)	1,8-Cineole	*Salmonella* sp.	Hayouni et al., 2008
78. *Sanguisorba minor* (Salad burnet)	Linalool, β-sitosterol	*E. coli, St. aureus*	Esmaeili et al., 2010
79. *Sassafras albidum* (Sassafras)	Safrole, Camphor,	*P. aeruginosa*,	Kamdem and Douglas, 2007
	Methyl eugenol	*S.* Typhimurium	Barbosa et al., 2012
80. *Satureja hortensis* (Summer savory)	Carvacrol, γ-terpinene, *p*-cymene	*B. subtilis, P. aeruginosa*,	Mihajilov-Krstev et al., 2010
		*C. albicans, S. cerevisiae*	
81. *Satureja montana*	Carvacrol, tannins, flavonoids,		Carraminana et al., 2008
(Winter savory)	triterpenes	*Ls. monocytogenes*	
82. *Schinus terebinthifolius*	Schinol, Quercetin	*St. aureus, B. cereus*	Carvalho et al., 2013
(Brazilian pepper)			Degaspari et al., 2005
83. *Sesamum indicum* (Sesame)	Latifonin, Momor-cerebroside,	*E. coli*	Ogunsola and Fasola, 2014
	Soya-cerebroside		Hu et al., 2007
84. *Sinapis alba* (White mustard)	Benzyl isothiocyanate	*E. coli*	Al-Qudah et al., 2011
	Benzyl nitrile, thymol		
85. *Smyrnium olusatrum*	Sabinene, Curzerene	———-	Mokaddem et al., 2010
(Alexanders)	α-Pinene, Cryptone		
86. *Syzygium aromaticum*	Eugenol	*E. coli, St. aureus*	Yadav and Singh, 2004
(Clove)		*S. anatum, B. cereus*	Naveena et al., 2006
		*C. freundii, K. pneumoniae*	Shan et al., 2007
			Sethi et al., 2013
87. *Tagetes minuta*	*cis*-β-ocimene	*E. coli, B. cereus, B. subtilis*	Sadia et al., 2013
	(Huacatay)	*St. aureus, Ps. aeruginosa, S.* Typhy	Senatore et al., 2004
		*C. albicans*	Shirazi et al., 2014
88. *Tasmannia lanceolata*	Polygoidal, Safrole,	*St. aureus*	Cock, 2013
(Tasmanian pepper)	Guaiol, Calamenene,	*E. coli, S.* Typhimurium	Weerakkody et al., 2010
	Myristicin, Drimenol	*Ls. monocytogenes*	
		*A. niger, C. albicans*	
89. *Thymus vulgaris* (Thyme)	Thymol, Cinnamaldehyde		Burt, 2004
			Jayasena and Jo, 2013
		*Ls. monocytogenes*,	
		*P. putida*	
90. *Thymus capitatus*	Thymol, Camphor,	*B. cereus, Salmonella* sp.	Boubaker et al., 2013
(Headed Savory)	Carvacrol	*Ls. innocua*	Bounatirou et al., 2007
91. *Thymus serpyllum*	Thymol, Carvacrol	*Ls. monocytogenes*	Skrinjar and Nemet, 2009
(Breckland thyme)		*St. aureus, E. coli*	Paaver et al., 2008
92. *Trigonella foenum-graecum*	Trigonelline	*E. coli, B. cereus*	Upadhyay et al., 2008
(Fenugreek)	Kaempferol 7-O-glucoside		Omezzine et al., 2014
93. *Trachyspermum ammi*	β-Phellandrene, α-Terpinene,	*C. albicans, Salmonella* spp.,	Khan et al., 2010
(Ajwan)	Limonene	*St. aureus, E. coli*	Chauhan et al., 2012
		*S.* Typhimurium	
94. *Vanilla planifolia*	Vanillin, Vanillic acid	*E. coli, B. cereus*	Menon and Nayeem, 2013
	(Vanilla)	*S. cerevisiae*,	Fitzgerald et al., 2003
		*Zygosaccharomyces bailii, Z. rouxii*	Shanmugavalli et al., 2009
95*. Verbena officinalis*	Citral, Isobornyl formate	*E. coli, S.* Typhimurium	Di Pasqua et al., 2005
(Vervain)		*Ls. monocytogenes, S. aureus*	De Martino et al., 2008
		*Lactococcus garvieae, L. plantarum*,	
		*L. delbrueckii*,	
		*Brochothrix thermosphacta*	
96*. Xylopia aethiopica*	4-Terpineol, 1,8-Cineole	*B. cereus, St. aureus*	Fleischer et al., 2008
(Grains of Selim)	Myrtenol	*P. aeruginosa, C. albicans*	Elhassan et al., 2010
			Vyry Wouatsa et al., 2014
97. *Zanthoxylum bungeanum*	Terpinen-4-ol, 1,8-Cineole,	*St. aureus*	Gong et al., 2009
(Chinese prickly ash)	Limonene	*B. cereus, B. subtilis*	Zhu et al., 2011
			Shan et al., 2007
98. *Zanthoxylum piperitum*	Sanshool	*St. aureus, E. coli*	Kim et al., 2007
(Japanese pepper)	*S.* Typhimurium		
99. *Zingiber officinale* (Ginger)	Gingerol, Shogoal,	*E. coli, Salmonella* spp.	Ghosh et al., 2011
	Methyl-isogingerol	Staphylococci, Streptococci	

## Importance of spices

Spices have been important to mankind since the beginning of history. Several mythological evidence including “Epic of Gilgamaesh,” and the “Bagavad Gita,” suggest their use for several purposes. Because of their strong preservative quality, spices were also used for embalming. According to Ayurveda, they help to maintain the balance of the body humors (Gupta et al., 2013a). Besides these, spices have been used to change the physical appearance of food. For instance, pepper and turmeric changed the color, appearance and the taste of food with many health benefits. Ginger, nutmeg and cinnamon improve digestion, considered good for spleen and sore throats (Prasad et al., 2011). Unfortunately, this beneficial effect of spices is not clinically proven. However, traditional practices emphasize the health benefits of spices. Eventually, recent studies highlighted other biological functions of spices, including antimicrobial, antioxidant, and anti-inflammatory (Tajkarimi et al., 2010).

## Spices for food preservation and safety

**Figure 1:**
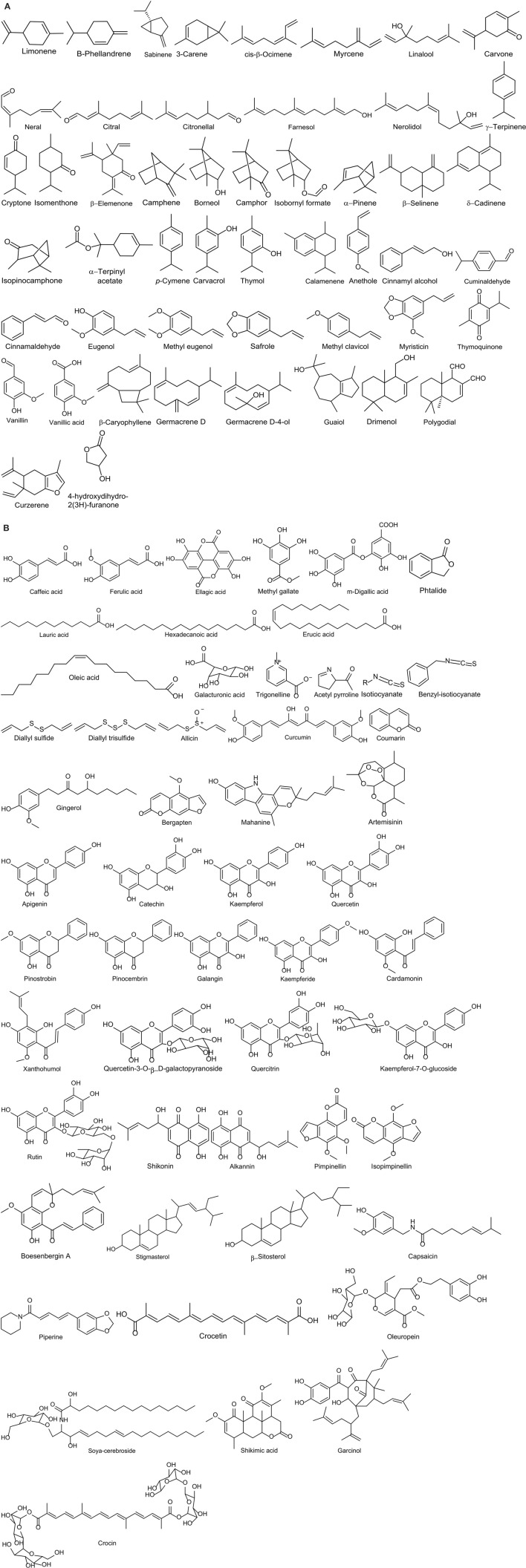
**Chemical structure of bioactive compounds of commonly used spices. (A)** Volatile compounds; **(B)** Not volatile compounds.

Food spoilage refers to an irreversible modification in which food becomes not edible or its quality is compromised. Such changes can be driven by different factors, either physical (oxygen, temperature, light) and/or biological (enzymatic activity and microbial growth). Despite the current technologies available in the production chain (for instance freezing, pasteurization, drying, preservatives), it seems impossible to eliminate completely the risk of food spoilage (Gutierrez et al., 2009). Lipid oxidation is one of the main issues of food spoilage. Hence, food industries have applied antioxidants such as butylated hydroxytoluene (BHT) and butylated hydroxyanisole (BHA) to prevent spoilage (Stoilova et al., 2007). However, their safety is doubtful and consumers are progressively demanding natural compounds. For this reason spices represent a potent tool for the food industry, thanks to their natural properties (Hyldgaard et al., 2012). Indeed spices possess antioxidant capacity, mainly due to the presence of phenolic compounds (Figures 1A,B). They exhibit antioxidant property by scavenging free radicals, chelating transition metals, quenching of singlet oxygen, and enhancing the activities of antioxidant enzymes (Rubió et al., 2013). Stoilova et al. (2007) reported that the CO_2_ extract of ginger had *in vitro* activity comparable with that of BHT in inhibiting the lipid peroxidation both at 37 and 80°C. Moreover, pimento and black pepper extracts reduced the formation of acrylamide up to 75 and 50%, respectively, in a model mixture simulating heated potato matrix (180°C for 20 min). Eugenol, the main component of pimento essential oil, limited the formation of acrylamide by 50% (Ciesarová et al., 2008). Some other studied antioxidants are: quercetine (dill), capsaicin (red chilli), curcumin (turmeric), carvacrol (oregano, thyme, marjoram), thymol (oregano, thyme), piperine (black pepper), gingerol, etc (ginger, marjoram; Figures 1A,B; Rubió et al., 2013; Przygodzka et al., 2014; Srinivasan, 2014). The relationship between antioxidant properties of spices and food spoilage has been well-documented.

Another issue in food spoilage is the microbial growth. Spices can also exert antimicrobial activity in two ways: by preventing the growth of spoilage microorganisms (food preservation), and by inhibiting/regulating the growth of those pathogenic (food safety; Tajkarimi et al., 2010). Studies regarding *in vitro* and *in vivo* antimicrobial activities of spices have been reported in the following sections.

### Antimicrobial activity *In vitro*

Numerous articles published in the last few decades have described the antimicrobial activities of spices *in vitro*. Extracts of entire plants, or part of them, obtained with diverse solvents (such as ethanol, methanol, ethyl acetate, and water) have been tested against microbes (Tajkarimi et al., 2010). Their essential oils or active compounds, alone or in combination, were also used to test the activity against different microbes (Singh et al., 2007; Weerakkody et al., 2010; Bassolé and Juliani, 2012). Disc-diffusion, drop-agar-diffusion, broth microdilution, and direct-contact technique in agar represent the most common methods utilized for screening (Tyagi and Malik, 2010a, 2011).

According to these reports, spices possess a very wide spectrum of activity against Gram-positive and Gram-negative bacteria, yeasts and molds (Tajkarimi et al., 2010; Table 1). Alves-Silva et al. (2013) reported that the bush-basil essential oils have antimicrobial activity against *Listeria innocua, Serratia marcenscens, Pseudomonas fragi, P. fluorescens, Aeromonas hydrophila, Shewanella putrefaciens, Achromobacter denitrificans, Enterobacter amnigenus, En. gergoviae*, and *Alcaligenes faecalis*, and against the yeasts *Yarrowia lipolytica, Saccharomyces cerevisiae, Candida zeylanoides, Debaryomyces hansenii*, and *Pichia carsonii*. Moreover, they were able to inhibit molds such as *Mucor racemosus* and *Penicillium chrysogenum*. In the same study, celery and coriander essential oils also showed a very similar antimicrobial activity against the tested strains.

Although the antimicrobial activity of spices may vary according to the types of spice (origin and bioactive compounds), different bacteria can react in different ways (Hyldgaard et al., 2012). Oregano essential oil showed higher antimicrobial activity against *Listeria monocytogenes* compared to *Escherichia coli* (Siroli et al., 2014b). Huacatay and basil essential oils were active against *Staphylococcus aureus* and *Bacillus subtilis* (Shirazi et al., 2014). Essential oil of angelica roots were effective against *Clostridium difficile, Cl. perfringens, Enterococcus faecalis, Eubacterium limosum, Peptostreptococcus anaerobius*, and in a lower extent against *E. coli* and *Bacteroides fragilis* (Fraternale et al., 2014). *Nigella sativa* extracts were more effective on *St. aureus* (5th day inhibition zone 34 mm) as compared to *E. coli* (5th day inhibition zone, 13 mm) and *P. aeruginosa* (5th day inhibition zone, 30 mm; Islam et al., 2012). *Rosmarinus officinalis* essential oil showed a strong antimicrobial effect against *Ls. monocytogenes* and *S. aureus* compared with *E. coli* (Jordán et al., 2013). A list of spices and their effects on most relevant bacteria is reported in Table 1.

Spices, essential oils and extracts have also been known for their anti-fungal activity (Table 1; Tajkarimi et al., 2010). Huacatay and basil essential oils were active against *Candida albicans* (Shirazi et al., 2014). Radwan et al. (2014) reported that among 22 common spice extracts, turmeric, and nutmeg extracts were the most active against different plant pathogens belonging to the genus *Colletotrichum.* In another study, where 23 spice extracts were studied, *Olax subscorpioidea* extract showed the highest antifungal activity, particularly against *C. albicans* and *C. tropicalis* (Dzoyem et al., 2014). A reduction of mycelial growth and inhibition of conidial germination and aflatoxin production by *A. flavus* were described by Nerilo et al. (2016) when 150, 10 and 15 μg/mL of ginger EO were applied, respectively. Ferreira et al. (2013) also reported a decrease (99.9 and 99.6%) of aflatoxin B1 and B2 when 0.5% of turmeric EO was employed while the same EO completely inhibited the biomass of *Fusarium graminearum* and its zearalenone production, at 3.5 and 3 mg/mL, respectively (Kumar et al., 2016).

Finally, antiviral activity of Mexican oregano against some viruses (i.e., acyclovir-resistant herpes simplex virus type 1 (ACVR-HHV-1), human respiratory syncytial virus (HRSV), and human rotavirus) has been reported (Pilau et al., 2011). Overall, it is difficult to predict how microorganisms are susceptible. In fact, spics constituents may impact several targets, such as microorganisms cell membrane, enzymes, and/or their genetic material (through the modulation of specific genes; Tajkarimi et al., 2010; Tyagi and Malik, 2010b,c; Hyldgaard et al., 2012).

#### Enhancement of the antimicrobial activity *in vitro*

To enhance the antimicrobial potential of spices or their constituents, the use of mixed extracts or natural compounds having different origins have been reported (Bassolé and Juliani, 2012). In most of the cases spices showed synergistic activities/effects. For instance, the antimicrobial activity of basil, oregano, bergamot, and perilla essential oils alone or in combinations, were tested. Basil and oregano essential oils alone had MICs of 1.25 and 0.625 μL/mL against *E. coli*, respectively, while their values were 0.313 μL/mL when used in combination. The MIC values against *St. aureus* for basil and bergamot EOs alone were for both 1.25 μL/mL, whereas the MICs of the two essential oils decreased to 0.313–0.156 μL/mL when combined, indicating higher antimicrobial activity. MICs of oregano and bergamot essential oils were 0.625 and 1.25 μL/mL against *B. subtilis*, respectively, whereas 0.313 μL/mL was determined for combined effect. Finally, the MIC values of oregano and perilla were 0.625 μL/mL for both against *S. cerevisiae*, while the mixture needed MICs of 0.313–0.156 μL/mL (Lv et al., 2011). In another study, Tabanelli et al. (2014) demonstrated the additive effect of citral and linalool against *S. cerevisiae*. In fact, linalool (250 mg/L) reduced markedly the amount of citral needed for the same effect (from around 150 to 50 mg/L). However, Tejeswini et al. (2014) reported antagonistic effects when cinnamaldehyde was combined with clove essential oils for molds inhibition.

The use of spice oils together with other preservation techniques has been also assessed. For example, low pressure atmosphere enhanced the susceptibility of *E. coli* and *S. enteritidis* to oregano, lemongrass or cinnamon essential oils *in vitro*. In particular, the MIC of cinnamon vapors for *S. enteritidis* decreased from 0.512 to 0.128 μL/mL (Frankova et al., 2014). Tabanelli et al. (2014) reported that the decrease of a_*w*_ potentiated the antimicrobial effect of citral (but not linalool) while lower pH favored the antimicrobial power of linalool (but not citral) against *S. cerevisae*. Some other hurdle technologies were also used for the enhancement of antimicrobial potential of essential oils. Tyagi and Malik (2010a, 2011, 2012) described the enhancement in antimicrobial potential of essential oils in combination of negative air ions (NAI) against food spoilage microorganisms.

### Antimicrobial potential in real food model system (*In vivo*)

Numerous natural compounds of spices with defined antimicrobial properties have been isolated. However, *in vitro* studies represent only one part of the use of active compounds as preservatives in food. Moreover, their physical and biochemical properties have been changed in real food systems due to the complexity of the food matrices (Tajkarimi et al., 2010). Therefore, whether spices or their components have the potential to inhibit the food spoilage and act as a food preservative has been determined in different studies.

As summarized in Table 2, the use of spices as preservatives has been assessed in multiple foods: meat, fish, dairy products, vegetables, rice, fruit, and animal food (Tajkarimi et al., 2010; Jayasena and Jo, 2013). Hernández-Ochoa et al. (2014) reported that cumin and clove essential oils inhibited the growth of total bacteria by 3.78 log CFU/g when used on meat samples for 15 days at 2°C. The antimicrobial activity of different spice extracts in raw chicken meat during storage for 15 days at 4°C was also studied. It has been found that the treatment of raw chicken meat with extracts of clove, oregano, cinnamon, and black mustard was effective against microbial growth (Radha et al., 2014). Essential oils of marjoram and coriander showed above 50% protection of chickpea seed from *Aspergillus flavus* infestation (Prakash et al., 2012). In an *in vivo* assay with cherry tomatoes (*Lycopersicon esculentum*), bay oil was effective against *Alternaria alternata* infection (Xu et al., 2014). In another experiment, Da Silveira et al. (2014) treated fresh Tuscan sausages with bay leaf essential oil. Comparing to the non-treated control, the essential oil was able to reduce the population of total coliforms (reduction of 2.8 log CFU/g) and extended the shelf life for 2 days. Rattanachaikunsopon and Phumkhachorn (2008) applied basil oil in *nham*, a fermented pork sausage, inoculated with *S. enteritidis* SE3 at 4°C. Basil oil reduced the number of bacteria from 5 to 2 log CFU/g after 3 days and the sensory evaluation suggested that these concentrations of oil were acceptable for the consumers. The isothiocyanates derived from oriental mustard reduced aflatoxins biosynthesis in *A. parasiticus* by 60.5–89.3% during Italian piadina storage (Saladino et al., 2016). Finally, Patrignani et al. (2015) reviewed the use of spices and their constituents in minimally processed fruits and vegetables.

**Table 2:** **Antimicrobial potential of phytochemicals (spices) for food preservation; *In vivo* study**.

**Scientific/Common name**	**Real food models**	**References**
1. *Allium sativum*	Prevent infections of *L. acidophlus, E. coli*	Yadav and Singh, 2004
	and *Aer omonas hydrophila* in poultry meat	
2. *Artemisia dracunculus*	Inhibit growth *St. aureus and E. coli* in cheese	Raeisi et al., 2012
3. *Boesenbergia rotunda*	Retard the growth of total viable counts of food pathogen	
	bacteria bacteria in Chinese sausage	Kingchaiyaphum and Rachtanapun, 2012
4. *Brassica nigra*	Reduce microbial growth in raw chicken meat	Radha et al., 2014
5. *Cinnamomum verum*	Potential bio preservative of banana, vegetables, dairy products	Sessou et al., 2012
	against *Aspergillus* spp., *Salmonella* spp.,	
6. *Citrus hystrix*	Inhibit the growth food pathogen bacteria in Chinese sausage	Kingchaiyaphum and Rachtanapun, 2012
7. *Ceratonia siliqua*	Inhibit the growth of *Ls. monocytogenes* in minced beef meat	Hsouna et al., 2011;
8. *Coriandrum sativum*	Protection of chickpea seed from *A. flavus* infestation	Prakash et al., 2012
9. *Cuminum cyminum*	Cumin seed oil protect stored protection of wheat	Kedia et al., 2014
	and chickpea against *Aspergillus* spp.	
	reduce total bacteria in meat samples	Hernández-Ochoa et al., 2014
10. *Cymbopogon citratus*	Inhibit the growth *B. cereus, S.* Typhimurium and *St. au reus*/	
	antibacterial agents in refrigerated chicken patties	Hayam et al., 2013
	control *Ls. monocytogenes* in bovine ground meat	De Oliveira et al., 2013
	inhibit microbial growth in real food system	Tyagi et al., 2013
		Tyagi et al., 2014a
11. *Cinnamomum cassia*	Raw chicken meat	Radha et al., 2014
	in Fresh sliced apples reduces natural microflora	Patrignani et al., 2015
	and inoculated *Ls. innocua*	
12*. Eryngium foetidum*	Reduce the growth of *Ls. monocytogenes* in pineapple juice	Ngang et al., 2014
13. *Laurus nobilis*	Bay essential oil reduce the population of total coliforms in fresh sausages	Da Silveira et al., 2014
	Protects cherry tomatoes against *Alternaria alternata* infection	Xu et al., 2014
14. *Mentha piperita*	*Mentha* essential oil inhibit *S. cerevisiae* growth in	Tyagi et al., 2013
	fruit (orange/apple) juice-potential natural food preservative	
15. *Olea europaea*	Antibacterial effect against *E. coli, P. aeruginosa, S. aureus* and	
	*K. pneumoniae in* shrimp/seafood industry	Ali et al., 2014
16. *Origanum vulgare*	Inhibit the growth of *L. monocytogenes, Aeromonas hydrophila*	
	and *E. coli* O157:H7 in meat, eggplant salad	Tajkarimi et al., 2010
	inhibition of *Pseudomonas* spp. in rabbit meat	Tajkarimi et al., 2010
	effectively inhibited the growth of *Salmonella* spp. in chicken meat	Burt, 2004
		Jayasena and Jo, 2013
	effective against microbial growth in raw chicken meat	Radha et al., 2014
	in Fresh sliced apples reduces natural microflora and inoculated *Ls. Innocua*	Patrignani et al., 2015
	Inhibit *E coli* O157:H7 in egg plant salad	Patrignani et al., 2015
	inhibit *Ls. monocytogenes, Y. enterocolitica*, and *A. hydrophilla* in Iceberg lettuce	Patrignani et al., 2015
	control the natural microflora and inhibit *Ls. monocytogenes*,	Patrignani et al., 2015
	*E. coli* in Lamb's lettuce	
17. *Origanum majorana*	Protection of chickpea seed from *A. flavus* infestation	Prakash et al., 2012
18. *Ocimum basilicum*	Inhibit the growth of *S.* enteritidis in fermented pork sausage	Rattanachaikunsopon and Phumkhachorn, 2008
19. *Piper nigrum*	Oil and oleoresins control microbial growth in orange juice	Kapoor et al., 2014
20. *Rosmarinus officinalis*	Inhibit the growth of *Ls. monocytogenes, Aeromonas hydrophila*	
	and *E. coli* O157:H7 in meat	Tajkarimi et al., 2010
	inhibition effect on *Ls. monocytogenes* in liver pork sausage	Tajkarimi et al., 2010
	inhibit *Ls. monocytogenes, Y. enterocolitica* and *A. Hydrophilla*	Patrignani et al., 2015
	in iceberg lettuce	
21. *Salvia officinalis*	Inhibit food spoilage in dairy products	Tajkarimi et al., 2010
	and *Salmonella* spp. in minced beef meat	Hayouni et al., 2008
22. *Satureja montana*	Control the growth of foodborne bacteria/improve quality of minced pork	Tajkarimi et al., 2010
23. *Syzygium aromaticum*	Inhibit the growth of *Ls. monocytogenes* in mozzarella cheese, meat	Tajkarimi et al., 2010
	and bovine ground meat	De Oliveira et al., 2013
	reduced total bacteria in meat samples	Hernández-Ochoa et al., 2014
	effective against microbial growth in raw chicken meat	Radha et al., 2014
24. *Thymus vulgaris*	Slight effect on *Ps. putida* in cooked shrimp sausages	Burt, 2004
	inhibit *E. coli* O157:H7 growth inhibition in lettuce and carrots	Patrignani et al., 2015
	and *L. monocytogenes* growth inhibition in minced pork	Burt, 2004
	control the natural microflora and inhibit *Ls. monocytogenes*,	Patrignani et al., 2015
	*E. coli* in lamb's lettuce	
25. *Thymus capitatus*	*Ls. monocytogenes* growth inhibition in minced beef meat	El Abed et al., 2014
26. *Zingiber officinale*	Potential biopreservative of beverages against food spoiling yeasts and bacteria	Sessou et al., 2012

Although several studies proved possible applications for spices and their derivatives as food preservatives, only few of them are currently applied on the market. For instance, rosemary is already employed for its preservative properties in meat products. Essential oil of rosemary has been used not only for its flavoring compounds but also for its antimicrobial and antioxidant activity. In fact, carnosic acid, one of its main component, is not only antimicrobial but it possesses an antioxidant activity higher than the common food additives, butylated hydroxytoluene (BHT), and butylated hydroxyanisole (BHA; De La Torre Torres et al., 2015).

Allyl isothiocyanate (AITC), a bioactive organosulfur compound found in cruciferous, plants, such as mustard, is known for its anticarcenogenic properties. It has been tested for effectiveness in preservation of fresh beef, sliced raw tuna and cheese. It possesses a strong antimicrobial activity against *E. coli* O157:H7, Salmonella enterica serovar Montevideo, *S. enterica* ser. Typhimurium, *P. corrugata, Campylobacter jejuni, St. aureus*, and *Ls. monocytogenes*. Moreover it has the generally recognized as safe (GRAS) status provided by the regulatory agencies of U.S. However, its application is sometimes limited because of its poor aqueous solubility, instability at high temperature, and susceptibility to degradation by nucleophilic molecules (Kim et al., 2002; Li et al., 2015).

#### Enhancement of the antimicrobial activity *In vivo*

Although some *in vivo* studies ended up with products acceptable for the consumers, the sensory aspect represents a critical point in the use of spices and their active compounds in food. In fact, sometimes MIC values were three or four times higher than those estimated *in vitro*, have been applied to have a measurable or stable antimicrobial effect *in vivo*. This aspect can dramatically affect the physical characteristics and organoleptic properties of the food products. To overcome these issues, several strategies have been exploited for the enhancement of antimicrobial potential of spices *in vivo*.

The synergistic effect of spices together with their constituents or other natural products has been tested. Water extracts of clove, cinnamon, and oregano were applied, alone (10 mg/L) or in combination (3.3 g/L each), in raw chicken meat and several characteristics were followed during storage for 15 days at 4°C. The mixture of the three extracts had the strongest impact on the bacterial load due to the synergistic actions of antimicrobial compounds present in the mixed spices (Radha et al., 2014). Siroli et al. (2014a) examined citral, carvacrol, citron essential oil, hexanal and 2-(E)-hexenal, alone (250 mg/L) or in combination (125+125 mg/L, except for the combination of citron essential oil/carvacrol, 200+50 mg/L, respectively), to sanitize minimally processed apples. The treatment with citral/2-(E)-hexenal and hexanal/2-(E)-hexenal maintained a good retention of color parameter within the 35 days and there were no yeast spoilage in any treated sample. Gabriel and Pineda (2014) studied the effect of different concentrations of vanillin and licorice root extract (LRE) on the mild heat decimal reduction times (D55-values) of a cocktail of *E. coli* O157:H7 in young coconut liquid endosperm. They found that the combined effect was most significant only at concentrations above 250 and 210 mg/L, respectively for vanillin and LRE. The efficacy of thymol (0.1% w/w) in combination with sodium lactate (1 and 2% v/w) was evaluated in fish patty samples stored at 4°C for 5 days. The presence of thymol plus 2% of sodium lactate had a synergetic effect against *S. enterica* ser. Typhimurium (Ilhak and Guran, 2014). Tejeswini et al. (2014) evaluated the antifungal activity of cinnamaldehyde, eugenol, peppermint, and clove essential oils and their combinations in tomato fruit system. While different concentrations of eugenol in combination with peppermint showed either additive or non-significant effect on mold inhibition, combination of cinnamaldehyde with clove essential oil produced non-significant or antagonist effects. Barbosa et al. (2014) also assessed the impact of basil essential oil alone or in combination with sodium hexametaphosphate (SHMP), on the shelf life of chicken sausage. Concentrations of 0.3 or 0.03% of essential oil inhibited the coliforms for 15 days at 4°C (*P* < 0.05). On the contrary, this effect was inhibited when SHMP was combined.

The synergistic effect of spices on other food preservation systems, such as mild thermal processing, has been also explored. Ngang et al. (2014) studied how to reduce the thermal impact during juice production. They demonstrated that pasteurizing pineapple juice at 60°C in presence of long coriander essential oil, lowered the time required for a 97% reduction of *Ls. monocytogenes* compared with treatment without essential oil. Similarly, mint, lemon grass, or eucalyptus essential oils worked synergistically with mild thermal treatment to inhibit the microbial growth in real food systems. Therefore, subsequent lower doses of oils were required for the food preservation (Tyagi et al., 2013, 2014a,b).

The use of spices together with additional high tech/cutting-edge technologies has also been studied. Pina-Pérez et al. (2012) demonstrated the applicability of Pulsed Electric Fields (PEF) in combination with cinnamon against *S. enterica* ser. Typhimurium to enhance the safety of dairy beverages. The maximum synergistic effect was achieved by 10 kV/cm–3000 μs PEF treatment with 5% (w/v) cinnamon. The maximum inactivation level (1.97 log_10_ cycles) was achieved at 30 kV/cm–700 μs plus 5% cinnamon. Patrignani et al. (2013) enhanced the effect of high-pressure homogenization (HPH) treatment (100 MPa for 1–8 successive passes) with citral into inoculated apricot juices, extending their shelf life in turn. Abriouel et al. (2014), instead, potentiated the effect of high hydrostatic pressure (HHP) on brined olives using thyme and rosemary essential oils. In other cases, novel technologies have been used to preserve the functional compounds. For instance, the use of AITC can be limited by its poor aqueous solubility, degradation by nucleophilic molecules, high volatility, and strong odor. Koa et al. (2012) masked the odor and volatility of AITC through its microencapsulation with Arabic gum and chitosan. In addition, Li et al. (2015) developed nanoemulsions that allowed a better aqueous solubility and chemical stability. Eventually, new packaging systems (active packaging) have been studied where essential oils or their main compounds were incorporated into the films. However, until now the research did not provide consistent results (Maisanaba et al., 2016). All these studies showed that the antimicrobial and food preservative potential of natural compounds can be enhanced or maintained by applying physical technologies.

## Mode of antimicrobial action of spices

Although the antimicrobial effects of spices and their derivates have been tested against a wide range of microorganisms over the years, their mode of action is still not completely understood. In fact, spices and their essential oils can contain many different bioactive compounds present in variable amounts. Basically, the bioactive constituents of spices can be divided into volatile and non-volatile compounds (Figures 1A,B). The first ones are mainly responsible for the antimicrobial activity of spices. They can be divided in four groups: terpens, terpenoids, phenylpropenes, and “others” (such as products of degradation; Hyldgaard et al., 2012). Terpens are evaluated as lesser active antimicrobial compounds amongst the other compounds. For instance, the weak activity of ρ-cymene, one of the main component of thyme, is mainly related to its action as a substitutional membrane impurity. It can affect the melting temperature and the membrane potential, which in turn causes a decrease in cell motility (Hyldgaard et al., 2012). On the other hand, terpenoids, such as the well-studied thymol and carvacrol, exert their antimicrobial activity due to their functional groups (hydroxyl groups and delocalized electrons). For instance, thymol can interact with the membrane both with the polar head-group region of the lipid layer, affecting the permeability, or with the proteins, determining an accumulation of misfolded structures (Hyldgaard et al., 2012; Marchese et al., 2016). These changes can lead to cell leakages that in turn can bring the cell to death (O'Bryan et al., 2015). Once it is inside the cells, thymol can also disrupt important energy-generating processes such as the citrate metabolic pathway and the synthesis of ATP (Hyldgaard et al., 2012; O'Bryan et al., 2015). Carvacrol acts mainly at the level of the membrane as a transmembrane carrier of monovalent cations, exchanging K+ with H+ in the cytoplasm (O'Bryan et al., 2015). Other organic compounds present in spices are phenylpropenes, such as eugenol and cinnamaldhehyde. The antimicrobial activity of eugenol is performed mainly at the level of the membranes and proteins, inducing permeabilization and enzyme inactivation. On the contrary cinnamaldheyde, although less powerful than eugenol, can react and cross-link with DNA and proteins other than interact with cell membranes. Eventually, spices possess other degradation compounds originating from unsaturated fatty acids, lactones, terpenes, glycosides, and sulfur- and nitrogen-containing molecues. For instance, the mode of action of AITC, a nitrogen-containing compound, is generally considered as a non-specific inhibition of periplasmic or intracellular targets. In fact, due to its highly electrophile central carbon atom, it can inhibit enzymes and affect proteins by oxidative cleavage of disulfide bonds (Hyldgaard et al., 2012). AITC is the main constituent of mustard essential oil. Clemente et al. (2016) reported that mustard EO induced cell cycle arrest, resulting in bacterial filamentation.

Other than affecting membrane and intracellular stability, Szabo et al. (2010) reported that clove, oregano, lavender, and rosemary essential oils possess quorum sensing inhibitory activity. For instance, molecules such as furanones can be internalized by bacteria, bind to LuxR-type proteins, and destabilize them (Camilli and Bassler, 2006). In this way spices could impact the motility, swarming, and biofilm production of bacteria. Overall, antimicrobial activity of spices cannot be confirmed based only on the action of one compound. The final activity is a synergistic effect of more components.

## Conclusion

Starting from the food preparation, spices can affect both food spoilage microorganisms (food preservation) and human pathogens (food safety) due to the antimicrobial and antifugal activity of their natural constituents. Spices are provided from natural herbs and plants and generally recognized as safe (GRAS) by the American Food and Drug Administration (FDA). However, the need of high amount of natural compounds represent the main limitation for effective performance against microorganisms. Mostly, their organoleptic characteristics may impact the results of *in vitro* and *in vivo* trials. For this reason, combinations of spices or their pure natural compounds, applied with or without additional technologies, represent a promising alternative to avoid this problem. Synergistic effects can lead to a reduction of both natural compounds used and treatment applied. In several cases, additive activities have been also reported. The study of spices, natural compounds, and novel combination technologies can be source of inspiration for developing novel or enhanced molecules acting against spoilage microorganisms.

## Author contributions

DG: Data compilation, manuscript writing, DB: Data compilation, table formation, SP: Data compilation, manuscript writing, and formating, AT: Data compilation, manuscript writing, editing and formatting, and final approval.

### Conflict of interest statement

The authors declare that the research was conducted in the absence of any commercial or financial relationships that could be construed as a potential conflict of interest.
